# A Maize *Necrotic Leaf Mutant* Caused by Defect of Coproporphyrinogen III Oxidase in the Porphyrin Pathway

**DOI:** 10.3390/genes13020272

**Published:** 2022-01-29

**Authors:** Yan Zhao, Wei Xu, Lijing Wang, Shuai Han, Yongzhong Zhang, Qingzhi Liu, Baoshen Liu, Xiangyu Zhao

**Affiliations:** 1 College of Life Sciences/State Key Laboratory of Crop Biology, Shandong Agricultural University, Tai’an 271018, China; zhaoyan183culb@163.com; 2 College of Agronomy/State Key Laboratory of Crop Biology, Shandong Agricultural University, Tai’an 271018, China; xwnxn2016@163.com (W.X.); 123456hanshuai@163.com (S.H.); zhangyz2005111@163.com (Y.Z.); liuqz1024@163.com (Q.L.); 3 College of Life Sciences, De Zhou University, Dezhou 253023, China; 18353700697@163.com

**Keywords:** maize, necrotic lesions, coproporphyrinogen III oxidase, light, temperature

## Abstract

Lesion mimic mutants provide ideal genetic materials for elucidating the molecular mechanism of cell death and disease resistance. The maize *necrotic leaf mutant* (*nec-t*) is a recessive mutant with necrotic spots and yellow-green leaves. In this study, we found that *nec-t* was a light and temperature-dependent mutant. Map-based cloning and the allelic test revealed that *nec-t* was a novel allelic mutant of the *Necrotic4* gene. *Necrotic4* encodes the coproporphyrinogen III oxidase (CPX1), a key enzyme in the tetrapyrrole pathway, catalyzing coproporphyrinogen III oxidate to protoporphyrinogen IX. Subcellular localization showed that the necrotic4 protein was localized in the chloroplast. Furthermore, RNA-seq analysis showed that the *Necrotic4* mutation caused the enhanced chlorophyll degradation and reactive oxygen species (ROS) response. The mechanism of plant lesion formation induced by light and temperature is not clear. Our research provides a basis for understanding the molecular mechanism of necrosis initiation in maize.

## 1. Introduction

Necrotic or *lesion mimic mutants* (*lmms*) exhibit spontaneous necrotic lesions on the leaves or stems without pathogen attack. Their phenotype is similar to the hypersensitive response (HR), which induces visible morphological variations through local programmed cell death (PCD) or cell necrosis [1,2]. *LMM genes* were first discovered in maize in the 1960s and subsequently reported in a variety of plants, such as maize [3,4,5], rice [6,7,8,9], Arabidopsis [10], and barley [11]. Over the past several decades, many LMM genes have been cloned, and it has been found that chlorophyll synthesis, fatty acid, lipid biosynthesis, riboflavin biosynthesis, and kinase signaling pathways are closely related to the formation of necrotic spots [3,12,13]. Although the heredity of plant lesion mimics obeys Mendel’s law, a large number of studies have found that its phenotype is susceptible to the influence of environmental factors and genetic background [14,15]. The occurrence time, size, and severity of lesion mimics are different under different environments or genetic backgrounds. Therefore, the biological mechanism responsible for plant lesion mimics is very complex. Identifying more lesion mutants and cloning-related genes is vital to elucidate how cell death is regulated and executed in plants.

Tetrapyrroles are critical compounds for multiple biological processes such as photosynthesis, respiratory metabolism, reactive oxygen species (ROS) scavenging, and oxygen transport. Chlorophyll, heme, siroheme, and phytochromobilin are four common kinds of tetrapyrroles in plants [16]. The synthesis of tetrapyrrole starts from glutamate, and from glutamate to uroporphyrinogen III is the same pathway for all the classes of tetrapyrroles. Then, uroporphyrinogen III is converted to protoporphyrin IX (proto IX, the direct precursor for both chlorophyll and heme synthesis) catalyzed by uroporphyrinogen decarboxylase (UROD), coproporphyrinogen III oxidase (CPOX), and protoporphyrinogen IX oxidase (PPOX) [16]. Subsequently, proto IX enters the heme branch and chlorophyll branch under the catalysis of iron chelatase (FeCh) and magnesium chelatase (MgCh), respectively. 

Most intermediate molecules of the tetrapyrrole biosynthetic pathways are photosensitizers, which produce high levels of singlet oxygen and toxic oxygen groups in the presence of light. So far, many genes responsible for lesion initiation involved in tetrapyrrole metabolism have been isolated in higher plants, and their mutants display a series of phototoxicity-related phenotypes [3,12]. The *Lesion22* (*Les22*) gene encodes uroporphyrinogen decarboxylase, a key enzyme in the tetrapyrrole biosynthetic pathway in maize. In *les22*, the significantly reduced activity of UROD leads to the accumulation of reactive oxygen species (ROS) in leaves, ultimately causing cell damage and plant death [3]. Mutations in maize *Camouflage1* (*Cf1*) [17] and Arabidopsis *Rugosa1* (*Rug1*) [18] encoding the porphobilinogen deaminase (PBGD) that functions in chlorophyll and heme biosynthesis all raised necrotic lesion formation and cell death. The *Ghlmmd* gene encodes 5-aminolevulinic acid dehydratase and is involved in lesion initiation in cotton [19]. The CPOX-deficiency mutant *lesion initiation 2* (*lin2*) in *Arabidopsis* [10], *rice lesion initiation 1* (*rlin1*) in rice [20], and *Glycine max lesion mimic mutant 2-1* (*Gmlmm2-1*) in soybean [21] all show lesion formation and a light-dependent cell death phenotype. All these reports suggest that lesion formation and tetrapyrrole biosynthesis are closely related.

The *necrotic leaf* mutant (*nec-t*) resulted from a natural inbred line 81,647, whose etiolated seedlings withered under normal growth conditions. Previous studies have confirmed that necrosis in *nec-t* was independent of biotic stresses and the mutant was controlled by a recessive gene localized to a 136.46-kb region on chromosome 2 [22]. This study identified *nec-t* as a novel allelic mutant of the *necrotic4*, which encodes the coproporphyrinogen III oxidase (CPX1). In *nec-t,* an 886-bp segment was inserted in the promoter region of *necrotic4*, significantly reducing the expression of *necrotic4*. Simultaneously, shading and temperature-sensitivity experiments confirmed that necrotic spots’ formation in *nec-t* depended on light and high temperatures. Thus, our study enriched our understanding of the mechanism of mimic lesion initiation.

## 2. Materials and Methods

### 2.1. Plant Materials and Growth Conditions

The *nec-t* is a natural mutant identified from the inbred line 81,647. The homozygous siblings of 81,647 were used as the wild type (WT) for experiments. The *nec4* (*nec4-N516B*) mutant was obtained from the Maize Genetics Stock Centre (https://chinese.maizegdb.org/data_center/stock; last accessed 8 September 2019). All materials were grown at the Experimental Station or State Key Laboratory of Crop Biology of Shandong Agricultural University in China.

### 2.2. Sequence Analysis

Predicted genes located within the candidate range were identified using the maize genetics and genomics database (Maize GDB) (http://www.maizegdb.org (accessed on 28 December 2021) Zea mays.AGPv4 B73). Specific primers (Supplementary Table S1) used to amplify the candidate genes in *nec-t* and WT were designed according to its genome sequence. Then, the PCR products of the candidate gene were subcloned into the TA Vector, followed by sequencing.

### 2.3. Allelism Analysis

The *nec-t* and *nec4* are recessive homozygous lethal mutants. By molecular marker identification, *nec-t*/+ plants and *nec4*/+ plants were selected for hybridization. F_1_ was obtained by the crossing of *nec-t*/+ with *nec4*/+. F_1_ individuals were planted and subjected to phenotypic assay. A segregation ratio of WT:necrotic mutant plants of approximately 3:1 would indicate that *nec-t* and *nec4* are allelic mutants; if all plants were WT, *nec-t* and *nec4* would not be allelic.

### 2.4. Determination of Chlorophyll Fluorescence Parameters

Chlorophyll fluorescence parameters were measured on the leaves of *nec-t* and WT plants during necrotic spot formation using a MINI-PAM portable chlorophyll fluorometer. The steady-state fluorescence (Fs) and maximum fluorescence (Fm) under natural light conditions were measured from 10:00 am to 12:00 am. In addition, minimum fluorescence (F_0_) and maximum fluorescence (Fm) under dark adaptation were measured after shading or dark treatment for 20 min. Five samples were collected in each group, and three biological replicates were carried out.

### 2.5. Leaf Shading Experiment

To carry out the shading experiments, *nec-t* and WT plants were planted in the field. The leaf’s central portion was shaded with silver paper before necrotic spots appeared, and the silver paper was removed to observe the occurrence of necrotic spots until a large number of necrotic spots appeared on the nonshielded parts of the leaves.

### 2.6. Temperature Sensitivity Experiment

The WT and *nec-t* were planted in a greenhouse with 12,000 LX light, 80% relative humidity, a 16-h light (day), and an 8-h dark (night) photoperiod. Then, plants were evenly divided into two groups and incubated in a light incubator at 22/24 °C (night/day) and 28/30 °C (night/day), respectively. The phenotypes of the two groups of seedlings were observed when necrotic spots developed in *nec-t*.

### 2.7. Photosynthetic Pigment Content Measurement

Seedlings were treated in the same way as in the temperature sensitivity experiments. Leaf samples were collected from the same parts of the WT and *nec-t* seedlings under different temperatures when the necrotic spots emerged in *nec-t* leaves. Approximately 100 mg of fresh samples was cut into small pieces and soaked in 95% ethanol at 4 °C in the dark for 48 h. After centrifugation, the supernatant was measured at 663 nm, 645 nm, and 470 nm with a UV-2450 instrument (Hitachi). The pigment contents were calculated using the method described by Arnon (1949) [23].

Chl a (mg/g) = [(12.7*OD663 − 2.69*OD645)*V]/(W*1000)

Chl b (mg/g) = [(22.9*OD645 − 4.68*OD663)*V]/(W*1000)

Caro (mg/g) = [OD470*(V/W) − 3.27*Chl a − 104*Chl b]/198

Each sample was assayed with three biological repeats.

### 2.8. Chlorophyll Synthesis Intermediates Measurement

Leaf samples were collected from the same parts of the WT and *nec-t* seedlings at 30 °C when the necrotic spots emerged in *nec-t* leaves. Approximately 300 mg of fresh samples was ground and extracted with 25 mL of 80% alkaline acetone (volume ratio of 80 acetone:20 ammonia solution (1%)). After centrifugation, the supernatant was measured at 575 nm, 590 nm, and 628 nm with a UV-2450 instrument (Hitachi). The contents were calculated using the formulas [24]:

ProtoIX (mg/g) = [562(0.18016*A575 − 0.04036*A628 − 0.04515*A590)*V]/(W*1000) 

Mg-ProtoIX (mg/g) = [584(0.06077*A590-0.01937*A575 − 0.003423*A628)*V]/(W*1000) 

Pchl (mg/g) = [611(0.03563*A628 + 0.007225*A590 − 0.02955*A575)*V]/(W*1000) 

Each sample was assayed with three biological repeats.

### 2.9. Transmission Electron Microscopy

Transmission electron microscopy assays were conducted as described previously [25]. Leaf tissues near necrotic spots of *nec-t* and WT at high temperature (approximately 7 days after germination) and at the same location of leaves in *nec-t* and WT at low temperature (about 10 days after germination) were sampled and soaked in 2.5% glutaraldehyde fixative solution followed by osmium tetroxide and then dehydrated in an ethanol series before being infiltrated with Spurr’s resin. Polymerization was performed at 70 °C for 8 h. The specimens were sliced to yield ultrathin sections, and stained with uranyl acetate and alkaline lead citrate before being examined with a JEM-1400Plus (JEOL, Tokyo, Japan) transmission electron microscope.

### 2.10. Histochemical Analysis

Hydrogen peroxide (H_2_O_2_) in leaves was detected by DAB staining, as described by Li et al. [26]. Seedlings and sample preparation were as described in the electron microscope observation experiment. About 5 cm leaf segments were dipped in DAB solution (1 mg/mL, pH = 3.8) and cultured in a growth chamber for 8 h, so that DAB was absorbed and reacted with H_2_O_2_. Subsequently, leaf segments were put in 75% ethanol and heated for 15 min, then transferred into 10% glycerol for microscopic examination (Olympus szx12, Tokyo, Japan).

### 2.11. RNA-Seq Analysis

Three biological samples per phenotype were collected from 10 plants/sample of the WT and *nec-t* at the two-leaf stage (before necrotic lesion appeared). The total RNA was extracted following the standard EasySpin plus Plant RNA Kit (Aidlab, Beijing, China) method. Library construction was performed using the Illumina HiSeq mRNA construction method and sequenced on the Illumina Hiseq 4000 platform at Novogene Bioinformatics Technology Co., Ltd. (Beijing, China). RNA-seq data were deposited in the National Center for Biotechnology Information (NCBI) Sequence Read Archive (SRA) under accession number SRP346340 (BioProject ID: PRJNA780769). Reads were aligned to the maize B73 genome (http://www.maizegdb.org/; last accessed 6 October 2021, Zea mays.AGPv4) using DESeq R package (version 4.1.1, USA) [27]. Data were normalized as reads per kilobase of exon per million fragments mapped, as the sensitivity of RNA-seq depends on the transcript length. Differential expression analysis of six samples was performed using the DESeq R package (version 2.1.2, USA), and p-values were adjusted to control the false discovery rate. Unigenes with an adjusted *p *(*q*)** value < 0.05 identified by DESeq were considered to be differentially expressed. GO annotation and GO enrichment analysis (*p *(*q*)** value < 0.05) of DEGs were performed to investigate their functions. GO enrichment analysis of the DEGs was conducted using GOseq R packages [28] based on Wallenius’ noncentral hypergeometric distribution. To further investigate the biological functions and interactions of genes, pathway-based analysis was conducted using KEGG [29].

### 2.12. Subcellular Localization

For subcellular localization of NEC-T (*Necrotic4*)**, the full-length CDS sequence of *NEC-T* was amplified by PCR from maize inbred line B73 and cloned into the transient expression vector pBWA(V)HS-NT-GLosgfp to generate the fusion genes NECT-GFP driven by a 35S promoter. The expression vector was introduced into the protoplast of maize. Fluorescence signals were detected using a Leica TCS SP5 II (Leica, Germany) laser scanning confocal microscope.

### 2.13. RNA Isolation and Quantitative Real-Time PCR

Total RNA was extracted from WT and *nec-t*, *nec4* using an EasySpin plus Plant RNA Kit (Aidlab, Beijing, China). High-quality first-strand cDNA was generated using a HiFiScript gDNA Removal cDNA Synthesis Kit (CwBiotech, Beijing, China). QRT-PCR was conducted using gene-specific primers. *ZmACTIN1* was employed as the standard internal gene to standardize the cDNA levels of target genes. The primers used in qRT-PCR experiments are listed in Supplementary Table S1. To test the expression profile of the *NEC-T* gene under different temperature conditions, leaves near necrotic spots in *nec-t* and *nec4* plants were collected.

## 3. Results

### 3.1. Necrotic Lesion Formation in NEC-T Depends on Light and High Temperature

**Figure 1:**
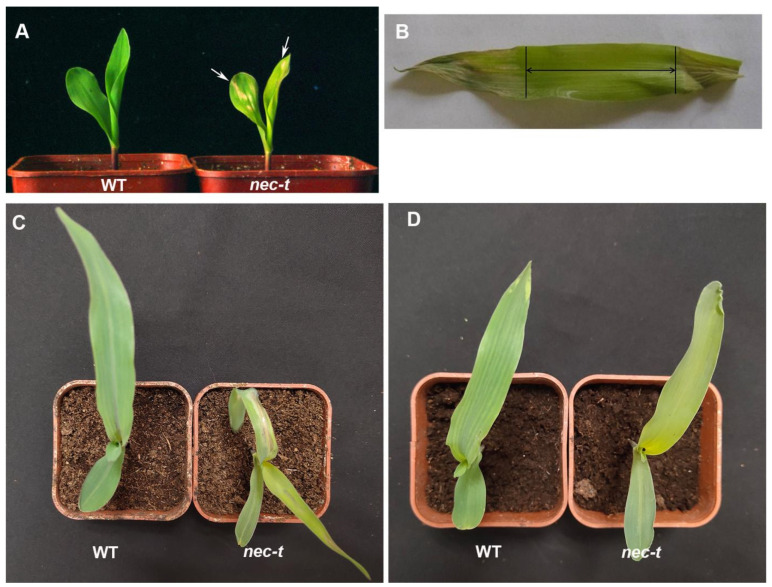
Requirement of light and high temperature for lesion development in *nec-t*. (**A**) The seedling phenotype of *nec-t* in the field. The arrowhead indicates necrotic spots. (**B**) Requirement of light for lesion formation in *nec-t* plant. The arrow indicates the leaf region wrapped using aluminum foil. (**C**) The phenotype of *nec-t* and WT plants (7 days) under high-temperature (30 °C) conditions. The arrowhead indicates necrotic lesions. (**D**) The phenotype of *nec-t* and WT plants (10 days) under low-temperature (24 °C) conditions.

*Necrotic leaf* (*nec-t*) was a spontaneous mutant identified from a maize inbred line 81647. In *nec-t*, leaves were yellow-green at the one-leaf stage, and irregular brown necrotic spots were initially visible along with the fully expanded leaves before spreading gradually over the whole leaf (Figure 1A). Previous studies have confirmed that the chlorophyll content of *nec-t* is significantly lower than that of WT [22]. We measured the chlorophyll fluorescence coefficient of *nec-t* and WT plants during the emergence of necrotic spots. The results showed that both the actual photochemical efficiency of PSII under light (ΦPSII) and the maximum photochemical quantum value PSII (Fv/FM) of *nec-t* were significantly lower than those of WT (Table 1), indicating that the light-harvesting complex function was damaged and the photosynthetic effect was reduced considerably.

Light was found to be the most important environmental factor inducing lesions formation. To confirm whether the formation of lesions in *nec-t* leaves was light-dependent, we covered the leaves with aluminum foil before necrotic spots appeared. Macroscopic lesions were formed on the exposed area, but no necrotic spots on the shaded area (Figure 1B), indicating that light exerts a deciding role in *nec-t* lesion formation.

Occasionally, homozygous *nec-t* plants exhibited few necrotic spots and could survive to produce seeds after being exposed to low temperatures for several days. Therefore, we speculate that the production of necrotic spots is also influenced by temperatures. To examine our hypothesis, WT and *nec-t* plants were planted in two illumination boxes under different temperatures. Consistent with the speculation, *nec-t* showed typical necrotic spots and eventually died when grown at 30 °C (Figure 1C). Although *nec-t* developed slowly and displayed a pale-yellow seedling at 24 °C, no necrotic spots were exhibited on *nec-t* leaves (Figure 1D), which suggested that the appearance of necrotic spots on *nec-t* depends on high temperatures. Meanwhile, the Chl a and Chl b contents in *nec-t* were higher under 24 °C but still significantly lower than those in WT (Figure S1A,B), and the chl a/b ratio was higher in *nec-t* than that in WT under 24 °C (Figure S1D). At the same time, it was found that, unlike under 30 °C, the carotenoid content in *nec-t* was not significantly different from that in WT under 24 °C (Figure S1C). This result suggested that the chloroplast structure of *nec-t* may be less damaged under 24 °C. Therefore, combined with above result, we determined that *nec-t* was sensitive to temperature, and high temperatures could induce necrotic spots.

### 3.2. Ultrastructural Observations of Chloroplasts at Different Temperature

**Figure 2:**
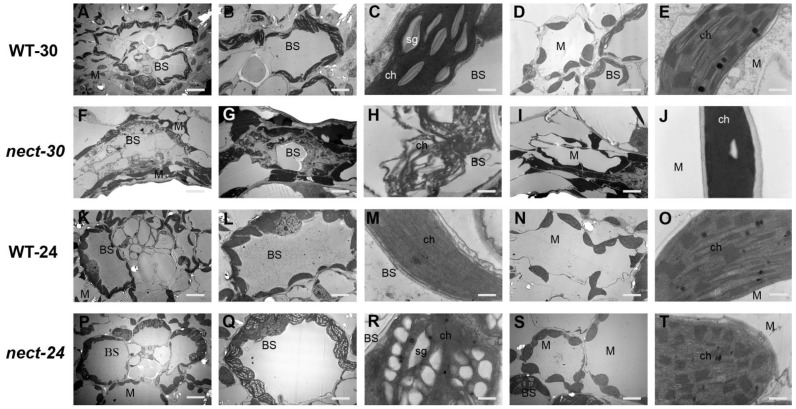
Chloroplast ultrastructure of the *nec-t* mutant and wild-type leaves at different temperatures. (**A**,**F**,**K**,**P**) Garland structure. (**B**,**G**,**L**,**Q**) Bundle sheath cells. (**C**,**H**,**M**,**R**) Chloroplast structure in vascular bundle sheath cells. (**D**,**I**,**N**,**S**) Mesophyll cells. (**E**,**J**,**O**,**T**) Chloroplast structure in mesophyll cells. WT-30 and *nec-t*-30 represent the wild-type and mutant tissues at 30 °C; WT-24 and *nec-t*-24 represent wild-type and mutant tissues at 24 °C. ch, chloroplast; sg, starch; BS, bundle sheath cells; M, mesophyll cells. Bar = 10 μm in (**A**,**F**,**K**,**P**), 5 μm in (**B**,**D**,**G**,**I**,**L**,**N**,**Q**,**S**), 500 nm in (**C**,**E**,**H**,**J**,**M**,**O**,**R**,**T**).

To characterize the influence of temperatures on cell necrosis, the chloroplast structure of WT and *nec-t* leaves at different temperatures were examined by electron microscopy. Under 30 °C and 22 °C, chloroplasts in WT leaves were normal: spindle-shaped and well-developed thylakoid (Figure 2A–E,K–O). Under 30 °C, the garland structure of necrotic leaves in *nec-t* was severely damaged (Figure 2F), accompanied by severely concentrated and degraded bundle sheath (BS) cells (Figure 2G,H) and mesophyll cells (Figure 2I,J). Under 24 °C, the garland structure of *nec-t* was complete, the chloroplast outer membrane of mesophyll cells was slightly damaged (Figure 2T), and significantly increased starch granules were seen in BS cells (Figure 2Q). According to these results, we conclude that *nec-t* chloroplasts are less damaged at low temperatures, which may be one of the reasons that *nec-t* can survive at low temperatures. 

### 3.3. High Levels of ROS Accumulated in NEC-T Mutant Leaves at High Temperature

**Figure 3:**
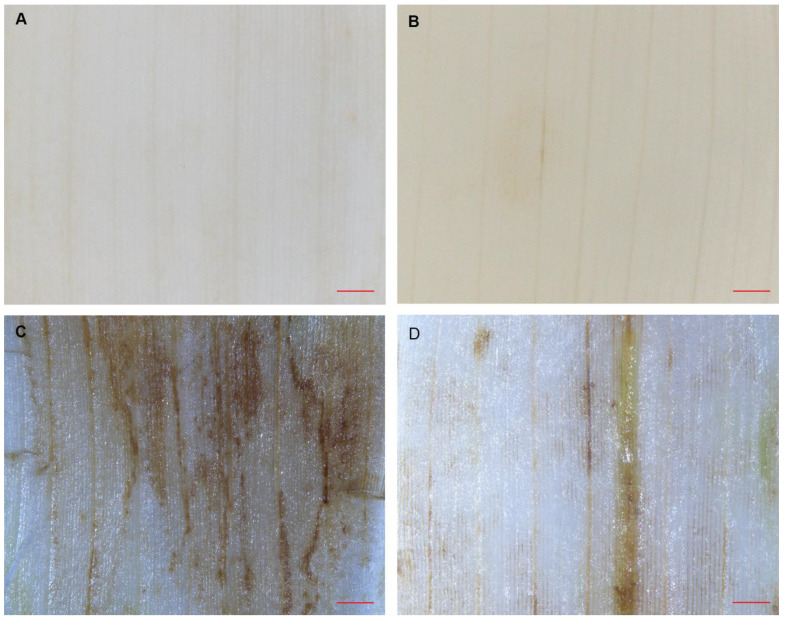
DAB staining for hydrogen peroxide of *nec-t* mutant leaves. (**A**,**C**) DAB staining of WT (**A**) and *nec-t* (**C**) leaves at 30°C. (**B**,**D**) DAB staining of WT (**B**) and *nec-t* (**D**) leaves at 24 °C.

The accumulation of high levels of ROS (e.g., H_2_O_2_) associated with chloroplast dysfunction and cell necrosis has been reported in many *Lmms* [6,12,17,21,30,31,32]. A terminal deoxynucleotidyl transferase-mediated dUTP nick end labeling (TUNEL) assay has confirmed that the cell death of *nec-t* leaf was not a programmed cell death and that a high level of ROS accumulated in *nec-t* [22]. The 3,3′-diaminobenzidine (DAB) staining was used to assess ROS in *nec-t* under different temperatures. As opposed to the *nec-t* leaves at 30 °C (intense brown staining showed in surrounding lesion sites) (Figure 3C), slight brown staining showed in the yellow-green leaf of *nec-t* at 24 °C, suggesting that a small amount of ROS was accumulated (Figure 3D). This result indicated that less low-temperature-induced ROS accumulated in *nec-t*, and this may be another reason why *nec-t* can survive at low temperatures. Alternatively, high ROS may cause chloroplast damages at high temperatures.

### 3.4. Determination of nec-t

**Figure 4:**
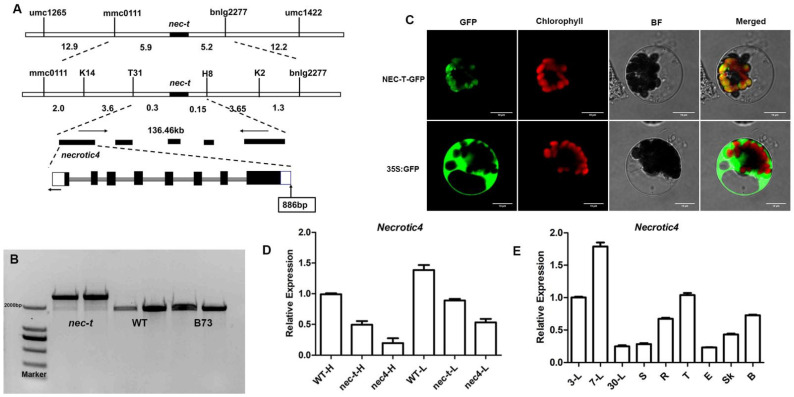
Map-based cloning and expression analysis of *nec-t*. (**A**) Map-based cloning of the *nec-t* gene. The black region represents the *nec-t* gene, and the arrow indicates the insertion position [22]. (**B**) Validation of inserted fragments in *nec-t*. The amplified fragment of WT and B73 was 2.0 k, and that of *nec-t* was about 2.9 k. (**C**) NEC-T protein is co-located with the chloroplast. NEC-T: the coding protein of *Nec-t* gene; GFP: green fluorescent protein; 35S: 35S promoter; NEC-T-GFP, NEC-T-GFP fusion protein; 35S:GFP: control of GFP protein. bar = 10 μm. (**D**) Relative expression of the *Necrotic4* gene under different temperatures in the leaves of WT, *nec-t*, and *nec4*. H, 30 °C; L, 24 °C. (**E**) Relative expression of the *Necrotic4* gene in different tissues. 3-L: Leaves of 3 days after germination; 30-L: Leaves of 30 days after germination; S: stem; R: root; T: tassel; E: ears. Error bars indicating SD were obtained from three biological repeats.

Previous studies have confirmed that the *nec-t* phenotype was controlled by a recessive gene localized to a 131.7-kb region on chromosome 2 according to the maize version 3 database (*Zea mays*. AGPv3 B73) [22]. According to the maize version 4 database, this interval is about 136.46 kb, and this study was based on the version 4 database (*Zea mays*. AGPv4). Based on the maize database, five open reading frames (ORFs) were identified within the mapped region (Table 2). By sequencing, we identified an 886-bp insertion located in the start site of *Necrotic4* (Figure 4A,B), while there was no difference in the other three genes, indicating that *Necrotic4* might be the target gene of *nec-t*. Amino acids sequence comparison found that *Necrotic4* encodes coproporphyrinogen III oxidase (CPX1), which catalyzes coproporphyrinogen III oxidate to protoporphyrinogen IX. Meanwhile, *Necrotic4* is the target gene of the *nec4* (*nec4-n516b*) mutant, which showed necrotic yellow-green seedlings: necrosis spots began from the leaf tips and then gradually spread to the whole leaves (Figure S2) [33]. The phenotype of *nec-t*/*nec4* was similar to *nec4* and the progenies of (*nec-t*/+)/(*nec4*/+) produced a 3:1 segregation of green and chlorophyll deficiency plants (273:94, χ2 = 0.07) (Figure S2). These results indicated that *nec-t* was a novel allele of *nec4,* and *Necrotic4* was the target gene of *nec-t.*

To assess the subcellular distribution of NEC-T, an expression vector containing 35S:NEC-T-GFP was transformed into maize protoplasts. Transient expression showed that the fusion proteins co-located with the chloroplast (Figure 4C). qRT-PCR analysis showed that the expression level of the *Necrotic4* gene in *nec-t* was significantly lower than that in WT at both temperatures, but the inhibition degree of *Necrotic4* at low temperature was significantly lower than that at high temperature (Figure 4D). In addition, the *Necrotic4* expression level in *nec-t* was higher than that in *nec4*, indicating that *nec-t* was a weak allele of *nec4* (Figure 4D). Tissue expression profile analysis showed that *Necrotic4* was constitutively expressed in all examined tissues (Figure 4E) and highest in the leaf, consistent with the expression pattern shown in the publicly available Maize Gene Expression database (qTell) (https://qteller.maizegdb.org/; last accessed 8 September 2021)(Figure S3).

### 3.5. Chlorophyll Synthesis in NEC-T Was Hindered

Protoporphyrin IX (*ProtoIX*), a porphyrin metabolite, is a direct product of chlorophyll synthesis. *NEC-T* encodes coproporphyrinogen III oxidase (CPX1) and catalyzes coproporphyrinogen III oxidate to protoporphyrinogen IX. Therefore, a decrease in CPX1 will lead to a decrease in *ProtoIX*, which in turn leads to a decrease in chlorophyll synthesis. Our results showed that the key intermediates (*ProtoIX, Mg-ProtoIX, Pchl*) in chlorophyll synthesis in *nec-t* were all significantly lower than those in WT (Table 3), which proved that the synthesis of chlorophyll was hindered. 

### 3.6. Transcriptome Analysis

**Figure 5:**
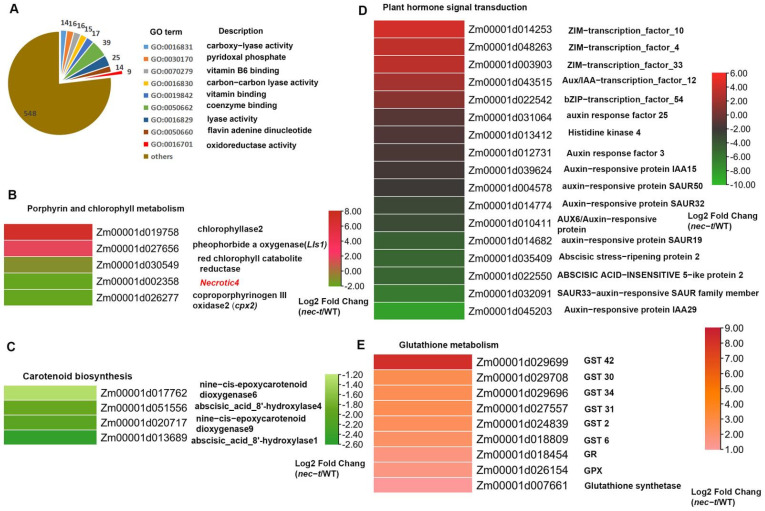
Identification of differentially expressed genes (DEGs) between WT and *nec-t* by RNA−seq. (**A**) The most significantly related GO terms of the functional annotated DEGs, *p *(*q*)** value < 0.05. (**B**) Heat maps of differentially expressed genes involved in porphyrin and chlorophyll metabolism. (**C**) Heat maps of differentially expressed genes involved in carotenoid biosynthesis. (**D**) Heat maps of differentially expressed genes involved in plant hormone signal transduction. (**E**) Heat maps of differentially expressed genes involved in ROS scavenging.

To better understand the impacts of the *nec-t* mutation on gene expression, RNA-seq was used to compare the transcript profiles in WT and *nec-t*. Among all the 30091 genes detected by RNA-seq, a total of 1392 genes showed a significantly altered expression between WT and *nec-t* (with 861 upregulated and 531 downregulated) (Supplementary File S1). Verification of the expression patterns of eleven DEGs via qRT-PCR revealed highly positive correlations between the RNA-seq data and qRT-PCR results (Supplementary Table S2). Among 1392 DEGs, 512 genes could be functionally annotated (BLAST against GenBank database at http://www.ncbi.nlm.nih.gov (accessed on 28 December 2021)). Based on GO analysis, these DEGs were classified into different biological processes and molecular functions, and the significantly enriched terms are shown in Figure 5A. KEGG analysis identified that 139 DEGs were enriched in 76 pathways, 10 of which were significantly enriched ( *p *(*q*)** value < 0.05). Some significantly enriched pathways are related to glutathione metabolism (zma00480), carotenoid biosynthesis (zma00906), porphyrin and chlorophyll metabolism (zma00860), and plant hormone signal transduction (zma04075).

According to RNA-seq results, we found two genes (*Zm00001d002358,* *Necrotic 4*; *Zm00001d026277, cpx2*) in the porphyrin pathway that were significantly downregulated, confirming that the synthesis of porphyrin was indeed affected in *nec-t* (Figure 5B). At the same time, two genes (*Zm00001d019758,* chlorophyllase 2; *Zm00001d027656*, pheophorbide a oxygenase, *Lls1*) involved in chlorophyll degradation was significantly upregulated, indicating that chlorophyll degradation was enhanced. Combined with the above results, we speculate that these differential expression genes directly lead to the decrease in protoporphyrin IX (decreased chlorophyll synthesis) and the enhancement of chlorophyll degradation, which finally leads to the decrease in chlorophyll accumulation and yellow-green leaf phenotype of *nec-t.*

KEGG analysis showed that carotenoid biosynthesis was the significantly enriched pathway for downregulated DEGs (Figure 5C). Carotenoids play an important role in light absorption as auxiliary pigments in photosynthesis. Violaxanthin and neoxanthin in the carotenoid biosynthesis pathway are the direct precursors of plant hormone ABA synthesis. The downregulation of multiple genes (Figure 5C) in this pathway resulted in the inhibition of carotenoid synthesis and decreased ABA content. Further analysis showed that the enriched DEGs in the plant hormone signal transduction pathway were mainly related to the ABA and IAA response, especially AUX/IAA proteins, ARFs, and SAUR proteins (Figure 5D). ABA and IAA can reduce the damage of free radical reactive oxygen species under stress conditions by increasing the activities of superoxide dismutase (SOD), peroxidase (POD), and catalase (CAT). Therefore, these results indicate that ABA and IAA responses to ROS are induced in *nec-t*.

Glutathione plays an important role in scavenging reactive oxygen species in plants. According to the RNA-seq results, we found that the gene expression levels involved in glutathione metabolism were mostly upregulated, especially glutathione S transferase (the main scavenger enzyme in the ROS degradation pathway) (Figure 5E). This corresponds to the results of histochemical staining: a large amount of H_2_O_2_ accumulation in *nec-t* (Figure 3). We speculate that in *nec-t*, the increased intracellular ROS activates the related ROS clearance genes. However, this reaction cannot balance the abnormal increase in ROS, which ultimately leads to cell death.

## 4. Discussion

Coproporphyrinogen III oxidase (CPO) is a key enzyme in the tetrapyrrole biosynthetic pathway that catalyzes coproporphyrinogen III (Coprogen III) to protoporphyrinogen IX (Proto IX). It has been shown that the defect of CPOX could also lead to the light-dependent lesion mimic phenotype in *Arabidopsis* [34], rice [20], and soybean [21]. In maize, there are two genes encoding coproporphyrinogen III oxidase, *cpx1* (*Necrotic4*) and *cpx2*. Except for the significantly different predicted N-terminal peptides in CPX1 and CPX2, the remaining amino acids in these two proteins are almost perfectly identical [33]. RNA-seq analysis showed that *cpx1* and *cpx2* were all downregulated in *nec-t*. At the same time, subcellular localization revealed that CPX1 was located in chloroplasts (Figure 4C), while CPX2 was predicted to be located in mitochondria [33] and the *cpx1* mutation was lethal. These results indicate that *cpx1* and *cpx2* did not have functional redundancy in maize.

In this study, allele analysis confirmed that the *necrotic leaf mutant* (*nec-t*) is a weak allele mutant of *nec4*, whose target gene (*Necrotic4)* encodes coproporphyrinogen III oxidase (CPX1). In *nec4*, necrosis spread inward from the leaf tip and covered the whole leaf, eventually leading to plant death (Figure S2). *cpx1*, another allele mutant of *nec4,* with a *Mu8* element inserted into the 5’end of exon 1 in the *Necrotic4* gene, displayed yellow shoots that quickly became necrotic upon exposure to light [33]. 

In *nec-t*, necrotic spots were randomly generated and gradually spread to the surrounding area exposed to light. Phenotypic differences in these mutants suggest that different sites of the *Necrotic4* gene have different functions. Chlorophyll is a common porphyrin compound in plants. In *nec-t*, the precursor content of chlorophyll was significantly lower than that of WT, and RNA-seq results showed that chlorophyll degradation was significantly upregulated, which may be the reason for the low chlorophyll content and yellow-green leaf phenotype. Previous studies have shown that the death of *nec-t* is not programmed cell death (PCD), but due to the accumulation of ROS. RNA-seq analysis revealed that, in *nec-t* mutants, multiple genes that participated in reactive oxygen scavenging were upregulated (Figure 5E). As the *Necrotic4* participated in the tetrapyrrole biosynthetic pathway and most of the intermediates in the tetrapyrrole biosynthetic pathways are photosensitizers, we speculate that the activation of ROS is due to the photooxidation of accumulated intermediate products (e.g., coproporphyrinogen III), and excess ROS led to the high expression of the gene involved in glutathione metabolism, but the response cannot completely relieve the damage caused by excessive ROS accumulation, ultimately leading to *nec-t* death.

Changes in light and temperature under natural conditions have a profound impact on plant growth and development [35]. Light, which can induce the production of radicals in the plant, is the most critical environmental factor for lesions formation. For example, rice *light-induced lesion mimic mutant* (*lil1*) [6], *lesion initiation 1* (*rlin1*) [20], *Arabidopsis catalase2* (*cat2*) [36], soybean *Glycine max lesion mimic mutant 2-1* (*Gmlmm2-1*) [21], maize *Lethal leaf spot-1(lls1)* [4], and *Spotted leaf 18* [37] are all light-dependent. Temperature is another important environmental factor affecting lesion mimic mutants’ phenotype. Usually, high temperatures can promote the formation of lesions. *Spl7,* the first cloned rice spotted leaf gene, encodes a heat stress transcriptional factor (HSF) with high-spot-density necrosis at higher temperatures [38]. The necrotic spots formation of the rice *spl7* mutant, which formed small, reddish-brown necrotic lesions in its adult plant, were enhanced by high temperatures [38]. At the same time, there is evidence that low temperatures are more likely to induce the formation of lesion mimics. *Arabidopsis* mutant *chilling-sensitive 4* *(chs4*) is a cold-sensitive mutant that is phenotypically normal at 20–22 °C, whereas its leaves become chlorotic and necrotic under cold stress [39]. In our study, we proved that *nec-t* was not only a light-dependent mutant but also a high-temperature-dependent mutant. The reasons for the difference of temperature effects need to be discussed. 

According to our results, we concluded that the excessive ROS in *nec-t* mainly comes from the photooxidation of tetrapyrrole, and the existence of light is the necessary condition for photooxidation. Therefore, the formation of necrotic spots in *nec-t* depends on the presence of light. The DAB staining experiment confirmed that the *nec-t* mutant accumulated more ROS at high temperatures, while only a small amount of ROS was accumulated at low temperature (Figure 3). In addition, compared with low temperature, chloroplasts were seriously damaged under high temperature, suggesting that chloroplast damage might be closely associated with the formation of necrotic lesions in *nec-t*. Meanwhile, the expression of *nec-t* is less inhibited at low temperatures (Figure 4D). Based on the above results, we speculated that necrotic spots did not occur at low temperatures, because *Necrotic4* was blocked to a low degree. Most of the ROS generated by the photooxidation of tetrapyrrole intermediates was removed by the ROS scavenging system. The residual ROS was insufficient to cause cell death, necrotic spots could not be generated, and the plants survived. However, the inhibition of *Necrotic4* expression still led to the reduction in chlorophyll synthesis, and *nec-t* seedlings were slightly yellowed due to insufficient chlorophyll.

*Necrotic* or *lesion mimic mutants* (*lmm*s) are ideal materials for studying the programmed defense response of plant cells. Although a large number of related genes have been cloned, the mechanism of lesion formation and the light- and temperature-induced necrosis of plants is poorly understood. We confirmed that *nec-t* is a yellow-green necrosis mutant dependent on light and high temperature due to mutations in *Necrotic4*. Different mutation sites of *Necrotic4* lead to incomplete identical phenotypes, which is helpful to improve our understanding of the mechanism of CPOX involved in plant development and cell death. Our findings also provide a basis for revealing the mechanism of light- and temperature-induced necrosis of plants.

## 5. Conclusions

The *nec-t* is a yellow-green necrosis mutant dependent on light and high temperature due to a defect of coproporphyrinogen III oxidase encoded by *Necrotic4* in the porphyrin pathway. *Necrotic4* plays a role in the chloroplast development and its mutation could enhance chlorophyll degradation and active oxygen accumulation.

## Figures and Tables

**Table 1:** Chlorophyll fluorescence parameters of *nec-t* and WT leaves.

	φPSⅡ	Fv/Fm
WT	0.441 ± 0.318 a	0.736 ± 0.363 a
*nec-t*	0.147 ± 0.021 b	0.627 ± 0.018 b

Small letters a and b indicate differences between *nec-t* and WT at *p* < 0.05, according to the least significant difference (LSD) tests. ΦPSII: the actual photochemical efficiency of PSII under light; Fv/Fm: the optimal/maximal photochemical efficiency of PSⅡin the dark.

**Table 2:** Predicted genes in the mapped region (136.46 kb) of *nec-t* gene.

Gene ID	Location	Gene Functional Annotation
*Zm00001d002358* (*Necrotic4*)	Chr2:10781211-10785395	coproporphyrinogen III oxidase
*Zm00001d002359*	Chr2:10788145-10793291	Probable potassium transporter 15
*Zm00001d002360*	Chr2:10838361-10839551	embryonic protein DC-8
*Zm00001d002361*	Chr2:10869880-10871152	Probable metal-nicotianamine transporter YSL7
*Zm00001d002362*	Chr2:10896918-10901679	methionine aminopeptidase

**Table 3:** ProtoIX, Mg-ProtoIX, and Pchl content in *nec-t* and WT leaves.

	ProtoIX (mg/g)	Mg-ProtoIX (mg/g)	Pchl (mg/g)
WT	0.873 ± 0.057 a	0.562 ± 0.039 a	0.504 ± 0.039 a
*nec-t*	0.370 ± 0.016 b	0.229 ± 0.012 b	0.180 ± 0.027 b

Small letters a and b indicate differences between *nec-t* and WT at *p *< 0.05**, according to the least significant difference (LSD) tests.

## Data Availability

The data presented in this study are available on request from the corresponding authors.

## Supplementary Materials

The following are available online at https://www.mdpi.com/article/10.3390/genes13020272/s1, Figure S1: Photosynthetic pigment content in the leaves of WT and *nec-t* mutant plants under different temperatures. Figure S2: Allelic complementation analysis. Figure S3: The expression pattern of *Necrotic4* from the publicly available Maize Gene Expression database (qTell). Table S1: Markers used in this research. Table S2: The expression value of 10 selected genes in RNA-seq and qRT-PCR. Excel S1: The DEGs data of *nec-t* leaves vs. WT leaves; Excel S2: KEGG enrichment of DEGs.
