# Supplementary material for: A Maize Necrotic Leaf Mutant Caused by Defect of Coproporphyrinogen III Oxidase in the Porphyrin Pathway
Source: Genes (Basel). 2022 Jan 29;13(2):272. doi: 10.3390/genes13020272 (PMC8872553; doi:10.3390/genes13020272)
Supplement: Supplementary file 1 [file genes-13-00272-s001.zip › Supplementary Figure.pdf]

Article title: A Maize *necrotic leaf mutant* Caused by Defective of Coproporphyrinogen III Oxidase in the porphyrinp athway

Authors: Yan Zhao[1,2], Wei Xu[2], Li Jing Wang[3], Shuai Han[2], Yong Zhong Zhang[2], Qing Zhi Liu[2], Bao Shen Liu[2*], Xiang Yu Zhao[1*]

Supplementary Figures

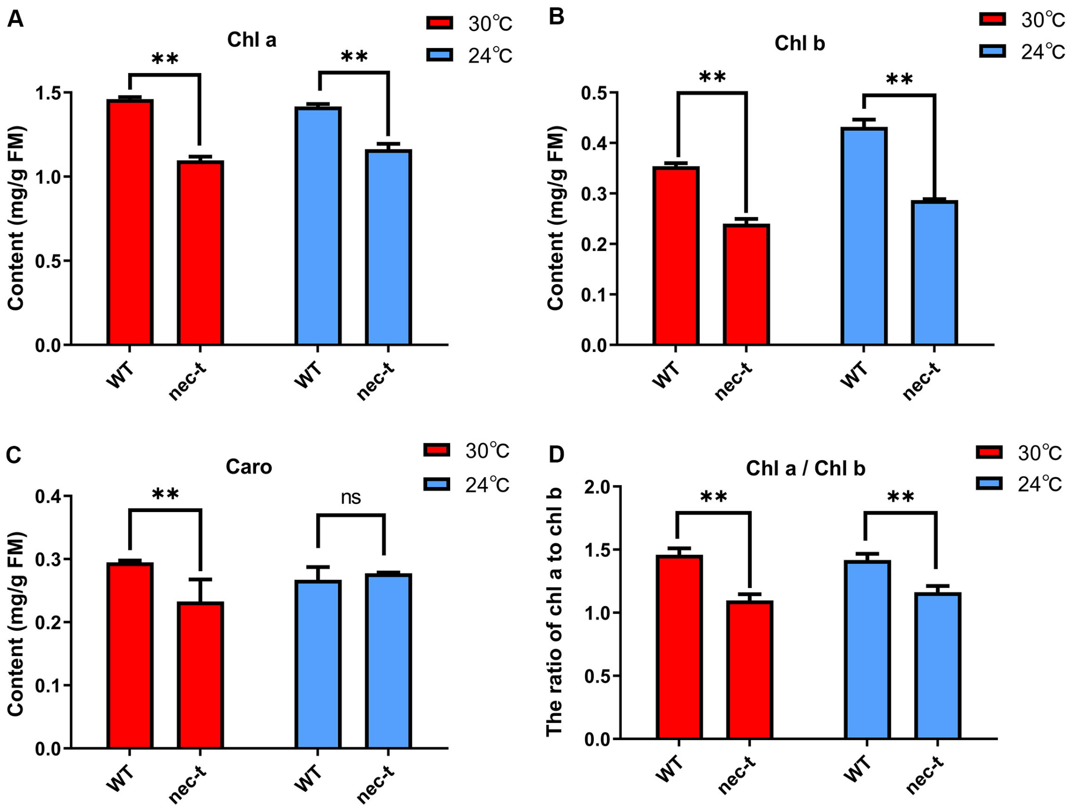

Supplementary Figure S1. Photosynthetic pigment content in the leaves of WT and *nec-t* mutant plants under different temperatures. (**A**) Chl a content at different temperatures. (**B**) Chl b content at different temperatures. (**C**) Caro content at different temperatures. (**D**) The ratio of chl a to chl b under different temperature conditions. Error bars indicating SD were obtained from three biological repeats. ** $P< 0.01$ (Student's t-test), NS $P> 0.01$ (Student's t-test).

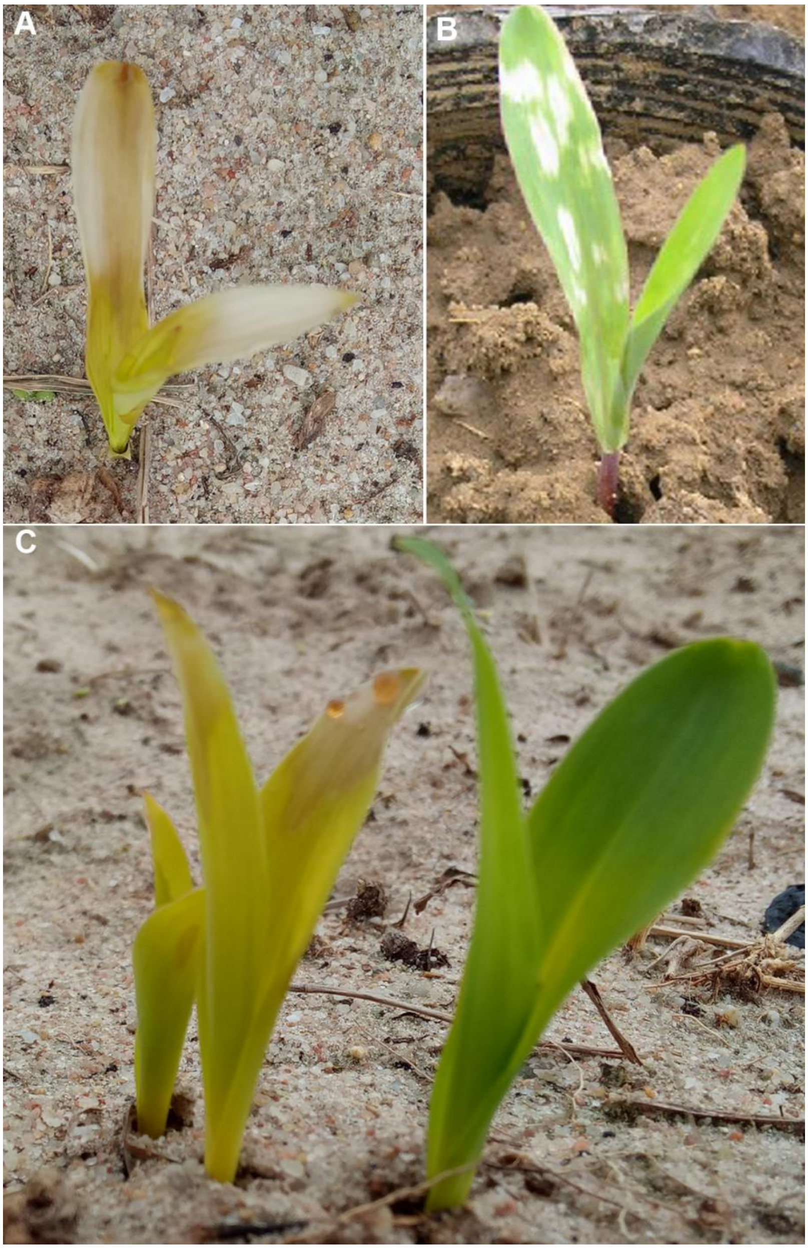

Supplementary Figure S2. Allelic complementation analysis. (**A**) Phenotype of the *nec4* mutant; (**B**) Phenotype of the *nec-t* mutant; (**C**) Phenotype of mutant plants from the filial generation produced from *nec4* and *nec-t* hybridization.

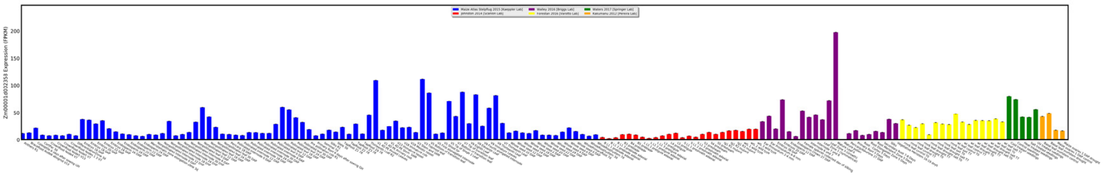

Supplementary Figure S3. The expression pattern of *necrotic4* from the publicly available Maize Gene Expression database(qTell). Different colors represent different processing conditions.
