# Supplementary material for: A Maize Necrotic Leaf Mutant Caused by Defect of Coproporphyrinogen III Oxidase in the Porphyrin Pathway
Source: Genes (Basel). 2022 Jan 29;13(2):272. doi: 10.3390/genes13020272 (PMC8872553; doi:10.3390/genes13020272)
Supplement: Supplementary file 1 [file genes-13-00272-s001.zip › Supplementary Tables.pdf]

Article title: A Maize *necrotic leaf mutant* Caused by Defective of Coproporphyrinogen III Oxidase in the porphyrinp athway

Authors: Yan Zhao[1,2], Wei Xu[2], Li Jing Wang[3], Shuai Han[2], Yong Zhong Zhang[2], Qing Zhi Liu[2], Bao Shen Liu[2*]， Xiang Yu Zhao[1*]

Supplementary Tables

Table S1. Markers used in this research.

Gene	Primers	Forward primer (5'–3')	Reverse primer (5'–3')
For *necrotic4* sequencing	*Nec4-1*	GGAGAGTTAGGGTTGAGTTGTTTATG	CGTGTCAGGTTAGGTTTTTTTGTTG
	Nec4-2	GCCGGACTGCAACACACGAC	CCACCAGAGAATGCCCAAGA
	Nec4-3	AGCTAGTCCCTCCTTTCTAATTTA	CACTGGGTCTGTTCTCTTTTTTT
	Nec4-4	GCCCACGCCGCTCATTA	CTTCACCCCAAGAACCCATT
	Nec4-5	CGGTGGCAGACACATTGAAG	GCCGGGCTATCGGAAGAA
For *Zm00001d002359* sequencing	*YS-1*	TCACTACTGCCTCAGCCATT	GTCCAGCGTCCATATTCTCA
For *Zm00001d002360* sequencing	*EP-P*	TGGTATTAACCGAGGAGCTG	AAGACACGTTGCGGTACAAG
	EP-1	AGCTTGTACCGCAACGTGTC	TGCATCTGCGATCAGGAGAG
For *Zm00001d002361* sequencing	*MA-P1*	TGCTCTCCGCTATAATGCAC	CCATTGACATCTCTACTCGG
	MA-P2	CCGAGTAGAGATGTCAATGG	GGACGAACAAGCAAGAGGA
	MA-1	TTAGTCCTTACCTACAGCGG	CATAGTCAAGAACACGAGCC
	MA-2	GCTCGTGTTCTTGACTATGC	GATCTTGCTGGTTCTTGTGG
	MA-3	CCACAAGAACCAGCAAGATC	AGAGAGTCATTGAACACCGC
	MA-4	CGGTGTTCAATGACTCTCTC	GCAAGCACTCTTCAGTAACC
	MA-5	GGTTACTGAAGAGTGCTTGC	AGGAGAACGTCACGAGACAT
For *Zm00001d002362* sequencing	*H15-1*	AAAACATCTCCTAAAACTCTCCC	CTTCCCACCTGATCATTAACTAC
	H15-2	GAGGATGATGAAGAGGAGGAG	TGGAACCAATGGAAGATGTAA
	H15-3	TATGTTTTGGTTGTCCTTTGGG	AACTATGGTGATTACCTGTTGCA
	H15-4	CATTATTTATTATTTTGGGAGGA	GAGATGTATGAACGATTTTGAG
	H15-5	CTTATTTTATGCTGGCTTTTCTT	TGTCTTTATACCCGTATCTGTGC

	H15-6	AGGTTCCTTTTCCAACGTGTATG	CTCGGTGTTTAGGCTTTTGTCTT
For qRT-PCR	*Nect-RT*	ATTGCGGAGAGGTCGTTATG	TAAGTGGAAGGGACACAAGTATG
	ACTIN	TCACCCTGTGCTGCTGACCG	GAACCGTGTGGCTCACACCA
	Zm00001d038806	GATGAAGTCGGACATCCTGATC	CTCCTCCATGATCCTCATCTTC
	Zm00001d039566	CTTCGGTTTCGTATGAACCATG	TGTCAGGATAACGTGTGTAGTC
	Zm00001d010411	CTATCCTTACACAGTCCGGTAC	TGTCCATAGACGATGTACCAAG
	Zm00001d022550	GCATTTGATTTCTTTGCAGGTG	AGTGGGACGACATTGTCTGAA
	Zm00001d012731	TTGCTCTAAAAGTACCGGCTAT	GCGATGAACAGAGTATTCACAC
	Zm00001d039624	TTTGTTTGAGAGTTCATGGCTC	AGTGTTACGACGAAACAAACAG
	Zm00001d008841	CTGTGCTTCTGAAACTTTGTGT	AGCAAAAACCCCAGTCGATATA
	Zm00001d031064	GTTTTTGTAAGCGCGAAAAGAC	TTCCCAAGAGAAGCTGGTTATT
	Zm00001d051556	GGCTTCAACATGATGCACTC	GATGCCGAGGTATCCGTAC
	Zm00001d020717	GGAGCTGAGGAAGAATTACTCC	CTGCATCGCCTTGTAATGGAG

Table S2. The expression value of 10 selected genes in RNA-seq and qRT-PCR.

Genes	RNA-seq		qRT-PCR		Gene name
	WT	*nec-t*	WT	*nec-t*	
Zm00001d038806	1	44.592	1	28.9	heat-shock_protein_101
Zm00001d039566	1	99.073	1	120.54	heat_shock_protein18f
Zm00001d010411	1	0.112	1	0.067	Aux/IAA-transcription_factor_38
Zm00001d022550	1	0.097	1	0.022	bZIP-transcription_factor_92
Zm00001d012731	1	0.343	1	0.166	ARF-transcription_factor_26
Zm00001d039624	1	0.291	1	0.207	Aux/IAA-transcription_factor_8
Zm00001d008841	1	222.86	1	117.90	heat_shock_protein18c
Zm00001d031064	1	0.486	1	0.075	ARF-transcription_factor_34
Zm00001d051556	1	0.027	1	0.019	nine-cis-epoxycarotenoid_dioxygenase6
Zm00001d020717	1	0.244	1	0.036	abscisic_acid_8'-hydroxylase4
